# A dural metastatic small cell carcinoma of the gallbladder as the first manifestation: a case report

**DOI:** 10.1186/s12957-018-1356-z

**Published:** 2018-03-16

**Authors:** Shuichi Tonomura, Tomoko Kitaichi, Rina Onishi, Yoshiaki Kakehi, Hisao Shimizu, Keiji Shimada, Kazuyuki Kanemasa, Akio Fukusumi, Nobuyuki Takahashi

**Affiliations:** 1 Department of Neurology, Nara City Hospital, 1-50-1 Higashi-kidera, Nara, Nara 630-8305 Japan; 2 Department of Gastroenterology and Hepatology, Nara City Hospital, 1-50-1, Kidera, Nara, Japan; 3 Department of Pathology, Nara City Hospital, 1-50-1, Kidera, Nara, Japan; 4 Department of Radiology, Nara City Hospital, 1-50-1, Kidera, Nara, Japan

**Keywords:** Gallbladder carcinoma, Meningioma mimics, Dural metastasis, Small cell carcinoma

## Abstract

**Background:**

A dural metastasis is one of the essential differential diagnoses of meningioma. In general, carcinomas of the breast and lung in females and prostate in males have been the most commonly reported primary lesions of dural metastases. However, dural metastasis of gallbladder carcinoma is extremely rare. Here, we report a unique case of a dural matter metastasis of gallbladder carcinoma as the first manifestation, which was autopsy-defined as small cell carcinoma.

**Case presentation:**

A 78-year-old man came to our hospital complaining of left hemianopia. Brain computed tomography (CT) revealed a sizeable parasagittal dural-based extra-axial tumor. However, the findings for meningioma were atypical by magnetic resonance imaging, suggesting a meningioma mimic. A contrast-enhanced CT scan of the abdomen revealed a large gallbladder carcinoma. The patient opted for the best supportive care and died 2 months later. The post-mortem examination revealed small cell carcinoma in gallbladder carcinoma. Moreover, an immunologically similar carcinoma was detected in the dural metastasis.

**Conclusions:**

To the best of our knowledge, this is the first case of a dural metastasis of gallbladder small cell carcinoma. A systemic examination is essential for clinicians when atypical findings of meningioma are observed, suggesting a meningioma mimic. We present this rare case with a review of the literature.

## Background

Meningioma is a common benign tumor that accounts for 20% or more of primary brain tumors [1, 2]. A dural metastasis mimics meningioma, although its treatment and prognosis can be different from meningioma. In general, carcinomas of the breast and lung in females and prostate in males have been the most commonly reported primary lesions of dural metastases [3, 4]. However, tumors from uncommon primary lesions with dural metastases have increasingly been cited. Gallbladder carcinoma, which has a high rate of lymph node or liver metastases, is a rare primary lesion of intracranial dural metastases. Small cell carcinoma (SCC) is a rare and highly aggressive feature of gallbladder carcinoma [5]. Here, we report a unique case of a dural metastasis of SCC of the gallbladder as the first manifestation and subsequently perform a literature review to consider the underlying mechanism.

## Case presentation

**Fig. 1:**
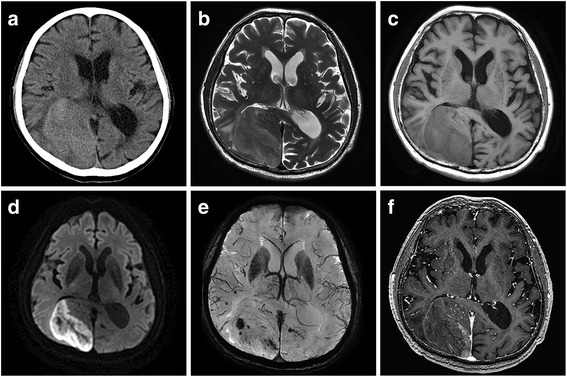
Axial brain CT and MRI. CT shows the extra-axial mass in the left posterior lobes (**a**). MRI shows hyperintensity on T2WI (**b**), hypointensity on T1WI (**c**), marginal hyperintensity on the diffusion-weighted image (**d**), intra-tumor hemosiderin deposits on T2*WI (**e**), and heterogeneous hyperintensity on gadolinium-enhanced T1WI (**f**)

**Fig. 2:**
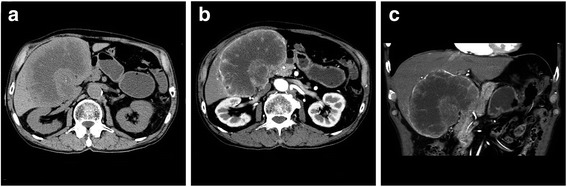
Axial abdominal CT. Large poorly marinated lobulated mass (**a**) with a heterogenous contrast enhancement (**b**, **c**)

A 78-year-old man was admitted to our hospital with the sole complaint of visual disturbance. There was no known past medical history or family history. He was a heavy smoker (more than 30 cigarettes per day) and a heavy drinker. On admission, a mass was identified in the right hypochondriac region by abdominal palpitation, and he had left hemianopia and topographical disorientation on neurological examination. Brain computed tomography (CT) showed a dural-based extra-axial mass, suggesting meningioma (Fig. 1a). The mass measured 50 × 55 × 65 mm. Brain magnetic resonance imaging (MRI) revealed hyperintensity on the T2-weighted image (T2WI) (Fig. 1b) and hypointensity on the T1-weighted image (T1WI) (Fig. 1c), which are also typical radiological findings of meningiomas. However, marginal hyperintensity on the diffusion-weighted image (Fig. 1d), hemosiderosis inside the tumor on the T2*WI (Fig. 1e), and heterogeneous enhancement on the gadolinium enhancement-T1WI (Fig. 1f) were atypical findings of meningioma. Therefore, we considered meningioma mimics and examined systemic disease, including a tumor, infectious disease, and non-infectious disease. Aspartate aminotransferase (85 IU/ml [normal, < 40 IU/ml]), alanine aminotransferase (55 IU/ml [normal, < 40 IU/ml]), and tumor markers, including carcinoembryonic antigen (133.1 ng/ml [normal, < 5 ng/ml]) and soluble interleukin-2 receptor (743 U/ml [normal, < 519 U/ml]), were all elevated. A non-contrast CT of the abdomen confirmed a poorly marginated, lobulated mass in the gallbladder fossa, with central necrosis (Fig. 2a). A contrast-enhanced CT demonstrated that the tumor was marginally enhanced (Fig. 2b) and was fed by multiple nutrient blood vessels, including the right hepatic artery. There was an invasion of the liver and engagement of the lymph nodes along the common hepatic artery, the hepatoduodenal ligament, and around the abdominal aorta. There was no ascites retention or pulmonary metastasis. Clinically, the patient was diagnosed with gallbladder carcinoma clinical stage IV according to the Union for International Cancer Control (UICC seventh edition), regardless of whether there was intracranial metastasis or not. The patient rejected a brain biopsy and fine needle aspiration. He opted for the best supportive care and died of the malignancy 2 months later.

**Fig. 3:**
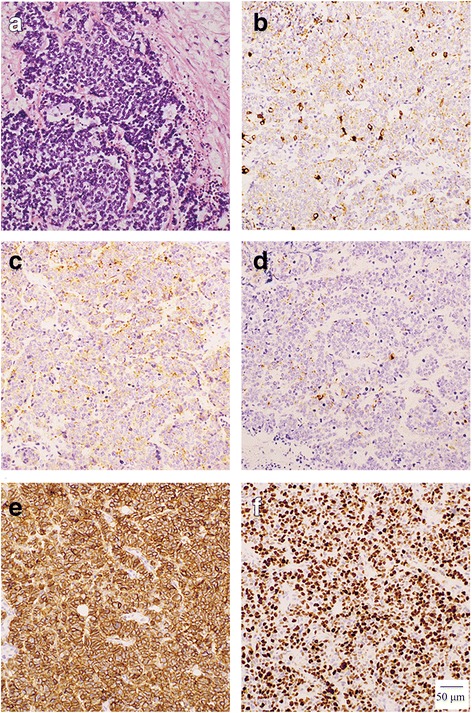
Small cell carcinoma (**a**) showing a trabecular and rosetta arrangement of small cells with extension necrosis (hematoxylin and eosin staining). The following positive immunostains were helpful for diagnosis **b** synaptophysin (SYN), **c** chromogranin A (CGA), **d** neural cell adhesion molecule (N-CAM), **e** cytokeratin AE/AE3, and **f** Ki-67

**Fig. 4:**
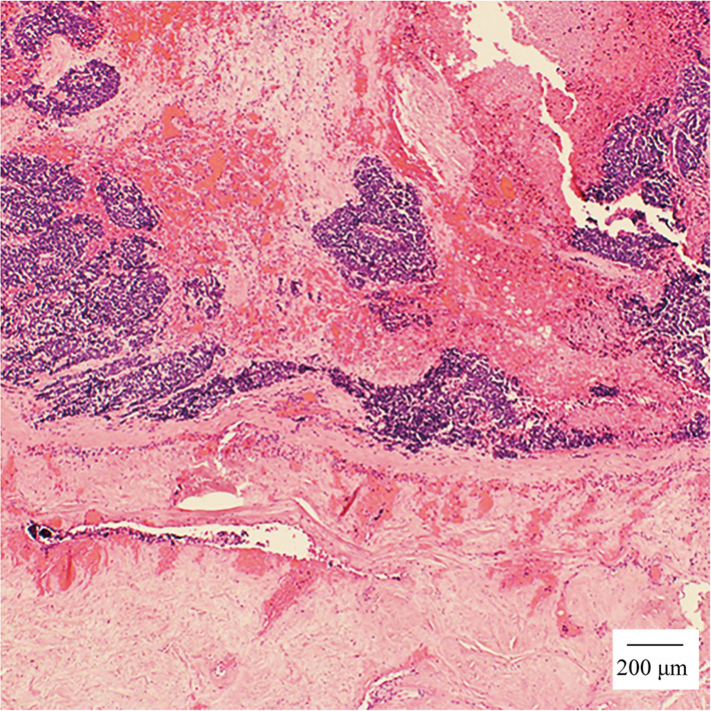
A trabecular and rosette arrangement of small cells with extension necrosis (hematoxylin and eosin staining) similar to a primary organ. There were tumor cells inside the dural vessels

An autopsy was conducted after written informed consent was obtained from the patient’s relatives. Macroscopically, the gallbladder tumor was yellow myxoid; it had spread to the liver and had adhered to the hepatoduodenal ligament, resulting in severe stenosis of the descending duodenum. Microscopically, the tumor had grown in diffuse sheets, was trabeculae, had a rosette arrangement of cells, and was extensively necrotic, as shown by hematoxylin and eosin staining (Fig. 3a). Tumor cells were small, with a round-to-fusiform shape, scant cytoplasm, finely granular nuclear chromatin, and absent or inconspicuous nucleoli. SCC of the gallbladder was diagnosed. Moreover, the diagnosis was immunologically confirmed using routine neuroendocrinal markers, including synaptophysin (Fig. 3b), chromogranin A (Fig. 3c), neural cell adhesion molecule (Fig. 3d), cytokeratin AE/AE3 (Fig. 3e), and Ki-67 (Fig. 3f). In the dura matter, SCC had invaded diffusely and some tumor cells were inside the dural vessels (Fig. 4).

## Discussion and conclusions

The liver and lymph nodes are the two most common sites of a metastasis of gallbladder carcinoma [6]. A brain metastasis of gallbladder carcinomas is relatively less common than other gastrointestinal carcinomas in both autopsied and clinical studies [7–9]. There are only a few cases of a dural metastasis of adenocarcinoma of the gallbladder in the literature [3, 10]. However, to the best of our knowledge, this is the first case of a dural metastasis of SCC of the gallbladder. Underlying mechanisms of a dural metastasis from primary lesions have been reported as a direct extension of a skull metastasis, and a bone-to-dura route and heavy lymphangitic and intravascular tumor burdens have been reported [4]. The bone-to-dura route was the most reasonable mode of propagation with breast and prostate carcinoma, which commonly metastasize to bone [11]. The hematogenous route was the most likely mode of propagation in other uncommon primary sites with a dural metastasis, including gastrointestinal, renal cell carcinoma, or skin cancers [12]. In our case, there were no overlying skull metastases or bony erosions based on clinical and autopsy findings. Moreover, a pathological diagnosis of SCC of the gallbladder, a rare and high-grade malignancy, tends to metastasize in the lymph node or liver [5, 13], suggesting a heavy lymphangitic and intravascular tumor burden.

In our case, CT showed the typical appearance of meningioma, which included a sharply demarcated and well-circumscribed lesion with an isodense-hyperdense gray matter. MRI also showed an average signal intensity; there was no signal intensity difference compared with cortical gray matter on T1WI, and it was iso-moderately hyperintense on T2WI. However, there was neither an intra-tumor diffuse enhancement, dural tail sign on gadolinium-enhanced T1WI, nor peri-tumoral edema on T2WI, which are atypical findings for meningioma. There are conflicting reports in the literature about imaging studies, including CT and MRI. In fact, it is challenging to distinguish a dural-based metastatic carcinoma from meningioma because it partially has the dural tail sign, vasogenic edema, and an intense contrast enhancement, which is a typical feature of meningioma [10]. Of the extra-axial tumors, atypical signal intensities on MRI images, a skull metastasis, or bony erosion due to a bone-to-dura tumor invasion may be useful to differentiate a dural metastasis from meningioma [10]. Because the prognosis in patients with malignancy harboring such an intracranial lesion is poor, clinicians must be aware of mimic meningiomas, even with unusual or atypical MRI features of meningiomas [14]. In conclusion, we present a unique case of a dural metastasis from SCC of the gallbladder. Even though it is an uncommon primary lesion, clinicians must be aware of a dural metastasis, even when there are some radiological findings that are typical of meningioma.

## Abbreviations

CT: Computed tomography
MRI: Magnetic resonance imaging
SCC: Small cell carcinoma
T1WI: T1-weighted image
T2WI: T2-weighted image
